# Guiacum in Dysmenorrhœa

**Published:** 1850-03

**Authors:** J. R. Stone

**Affiliations:** Newton, Illinois


### ARTICLE VI.

*Guaiacum in Dysmenorrhœa.*—By Dr. J. R. Stone, of Newton, Illinois.

I have in two instances, upon the same individual, used this article, in substance, in the treatment of acute symptoms in the above disease, with the happiest effect.

The patient was unmarried, twenty-five years of age, plethoric, and robust; had for years labored under an obstruction to the menstrual flow, which, at each monthly period, subjected her to convulsive attacks of the most alarming character.

The more dangerous symptoms were preceded by an acute lancinating pain in the epigastric and left lumbar regions, which augmented to such a degree as to deprive the patient of consciousness, and cause violent muscular spasms, amounting in some instances, to protracted opisthotonos. This state was accompanied by coldness of the extremities, turgid face, rolling of the eyes, imperceptible pulse, and trismus.

I was consulted in regard to her case, a short time subsequent to one of these attacks, and was of the opinion that the disease was in part attributable to malformation, as well as functional derangement of the organs engaged in the menstrual process. The discharge at each period had been quite limited, and showed an entire absence of healthy color.

To correct the latter difficulty, I commenced the exhibition of the Precipitated Carbonate of Iron, [Braithwaite's Retrospect for Nov.,] and although upon being called to attend the patient at the subsequent period, I found dangerous symptoms attending the menstrual effort; still it was gratifying to learn that the secretion had assumed a healthy hue.

At the most severe period in the attacks, the state of the pulse precluded bloodletting, which was verified by opening a vein without effect; I therefore resorted to Opium and Æther, the administration of which was followed by an abatement of dangerous symptoms, although the local pains remained excruciatingly severe. Blisters applied to the above mentioned regions, caused a metastasis to the back and shoulders.

At this period, depletion had usually been resorted to, in former treatment, which, in most instances, gave only partial relief; while convalescence was protracted to a discouraging length of time. I have no doubt that a majority of the profession, would, in this case, resort to a direct depletion; the patient, as I stated, being plethoric and remarkably robust: but in its stead I resolved to test the powers of Guaiac, in increasing the secretions, and accordingly ordered the administration of the following electuary, at intervals of three hours, and before the third one had been taken, every symptom of indisposition had disappeared:

℞ Pulv. Guaiaci Res.,
" Gum Arabic, a a gr. xxxv.,
" Cinnamon, gr. x.,
Sach. Alb., ℥ ss.
Water sufficient to form a consistent electuary.

In another case of acute Amenorrhœa from cold, complete restoration followed the administration of the following powder, at intervals of three hours, ere the third one had been taken; and I determined to try their effects, upon the patient under consideration, should an opportunity afford:

℞ Pulv. Rad. Colch.,
" Ergot,
" Cinnamon, a a gr. viij.

The following period brought forth an attack as severe as usual, and Opium, Æther, Asofœtida, &c., failing to moderate the symptoms, I with some difficulty succeeded in the administration of an emetic of Ipecac, gr. xxx, which had the desired effect, and after waiting an unnecessary length of time, for the action of the above powders, which had been given, but which failed of producing any change, I ordered the electuary, which produced prompt and rapid convalescence.

I have been thus prolix in the description of this case, and its treatment, not so much from its importance *per se* in a medical point of view, as to elicit information in regard to the therapeutic virtues of Guaiac.

Nothing need be feared from the magnitude of the dose in cases of the above character; and the indications in its administration are to augment such secretions, as by their deficiency or retention are likely to be the source of the disease.

Any information in possession of any of the profession in regard to its qualities, not hitherto made known, is solicited.

